# Effects of urban green infrastructure (UGI) on local outdoor microclimate during the growing season

**DOI:** 10.1007/s10661-015-4943-2

**Published:** 2015-11-07

**Authors:** Yafei Wang, Frank Bakker, Rudolf de Groot, Heinrich Wörtche, Rik Leemans

**Affiliations:** Environmental System Analysis group, Wageningen University, P.O. Box 47, 6700 AA Wageningen, The Netherlands; INCAS³, P.O. Box 797, 9400 AT Assen, The Netherlands

**Keywords:** Green infrastructure, Urban microclimate, Outdoor thermal comfort, Plant area index, Field measurements

## Abstract

This study analyzed how the variations of plant area index (PAI) and weather conditions alter the influence of urban green infrastructure (UGI) on microclimate. To observe how diverse UGIs affect the ambient microclimate through the seasons, microclimatic data were measured during the growing season at five sites in a local urban area in The Netherlands. Site A was located in an open space; sites B, C, and D were covered by different types and configurations of green infrastructure (grove, a single deciduous tree, and street trees, respectively); and site E was adjacent to buildings to study the effects of their façades on microclimate. Hemispherical photography and globe thermometers were used to quantify PAI and thermal comfort at both shaded and unshaded locations. The results showed that groves with high tree density (site B) have the strongest effect on microclimate conditions. Monthly variations in the differences of mean radiant temperature (∆Tmrt) between shaded and unshaded areas followed the same pattern as the PAI. Linear regression showed a significant positive correlation between PAI and ∆Tmrt. The difference of daily average air temperature (∆*T*_*a*_) between shaded and unshaded areas was also positively correlated to PAI, but with a slope coefficient below the measurement accuracy (±0.5 °C). This study showed that weather conditions can significantly impact the effectiveness of UGI in regulating microclimate. The results of this study can support the development of appropriate UGI measures to enhance thermal comfort in urban areas.

**Electronic supplementary material:**

The online version of this article (doi:10.1007/s10661-015-4943-2) contains supplementary material, which is available to authorized users.

## Introduction

The global urbanization process accelerates population growth in cities, causing changes in the urban microclimate, and ultimately affects people’s health. Although the mitigation of urban warming and amelioration of thermal comfort by urban green infrastructure (UGI) have been well established (Taha et al. 1991; Akbari et al. 2001; Yang et al. 2011; Ng et al. 2012; Berry et al. 2013; Skoulika et al. 2014), the underlying mechanisms and quantitative effects on the microclimate and human perceptions are still poorly understood. In an earlier study (Wang et al. 2014), we summarized the factors that possibly influence the performance of microclimate regulation by UGI, which are vegetation characteristics (quality and quantity, plant area index (PAI) (PAI = leaf area index (LAI) + wood area index (WAI)), vertical structure, species composition), local morphology (distribution of buildings and vegetation), ground properties, and weather conditions. However, it is less clear how strongly these factors affect the vegetation’s performance on microclimate regulation in different times of the year.

In our previous field study (Wang et al. 2015), temperature and humidity were continuously measured in a small local urban area during summer and winter and combined with numerical modeling. A significant effect of the local morphology as well as weather conditions on microclimate regulation by UGI was found, and vegetation density and PAI of trees seemed to strongly affect the microclimate regulation capacity (Wang et al. 2014). However, the relation between the vegetation’s PAI and the surrounding thermal environment was mainly analyzed by applying models (Shashua-bar and Hoffman 2000; Ng et al. 2012; Shahidan et al. 2012). To examine the impact of vegetation characteristics in terms of vegetation density and PAI on the capacity of UGI to regulate microclimate, field measurements were conducted at different locations covered by various types and configurations of green infrastructure during the growing season (from April to August 2014) to quantify the effects of changes in PAI and to provide empirical evidence. In addition, the effects of weather conditions on the microclimate regulation performance of UGI during the growing season were measured and are presented and discussed in this paper.

## Materials and methods

### Site description and field measurements

#### Site description

**Fig. 1:**
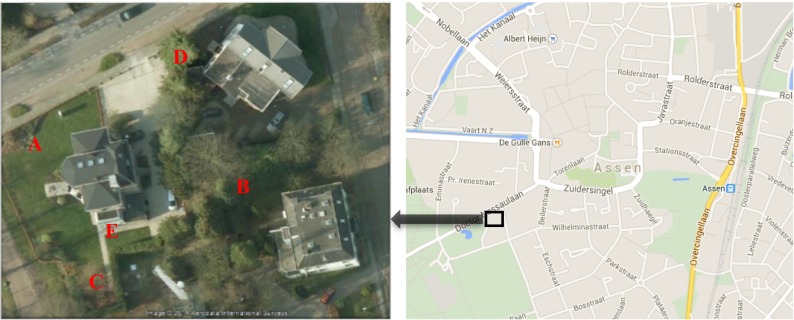
The location of the observation sites in the city of Assen, The Netherlands. Source: Google Earth (*left*) and screenshot of OpenStreetMap (OSM; *right*)

Assen, the capital of the Drenthe Province, is located in the north-western part of The Netherlands. It mainly enjoys a typical oceanic climate with mild winters and cool summers. May and June (spring) are the sunniest months of the year, and July and August (summer) are the warmest months. In the autumn, the weather becomes cooler, cloudy, and rainy, with frequent winds, while the sunshine time is only around 2 h each day. During this period, microclimate regulation by UGI is less important with less shade and evapotranspiration and does not play an essential role in human thermal comfort. Therefore, our study was targeting on spring and summer, and the measurements were carried out during the growing seasons, which were from leafless season (April and May) to full leaf season (June to August). We have conducted field measurements at five sites in a small urban area (approximately 3600 m^2^, latitude of 53° 0′ 0″ N, longitude of 6° 55′ 00″ E) in Assen (Fig. 1). The microclimatic data obtained from the study site’s open space (site A) supplied the reference data to compare with the other observation sites. This particular site was selected because of its best sunlight exposure, which minimized the influence from the surroundings. Different types of UGI were included in the study area: site B was surrounded by a group of trees (situated in a grove), site C was beneath a single deciduous tree, site D was located at the street side to observe the impact of street trees, and site E was placed adjacent to the building to determine the effect of the building’s façade on ambient microclimate.

#### Field measurements

Temperature and humidity stations were mounted at a height of 1.5 m. To observe variations in the effects of trees on the microclimate in the growing season, air temperature (*T*_*a*_) and relative humidity (RH) were consistently measured at all five sites from April to August 2014 (a 134-day period). Besides, globe temperature (*T*_*g*_) and wind velocity (*V*_*a*_) were also measured in sites A and B to examine outdoor thermal comfort (predicted mean vote (PMV) or physiological equivalent temperature (PhET) value) with and without surrounding vegetation, respectively. In this study, Va was continuously measured by a Davis Anemometer. However, technical limitations of the globe thermometer (globe diameter 75 mm) allowed us to measure Tg only on 23 clear, sunny days throughout the observation period. All data was acquired with a 10-min interval and stored in a dedicated database.

To observe the variations in a tree canopy, hemispherical images were taken during the growing seasons at all observation sites using a Panasonic Lumix DMC-FZ100 digital camera equipped with a Panasonic super fisheye wide-angle lens. Because sites A and E were located outside vegetative canopies and site D was only shaded by evergreen trees where the changes of a canopy are negligible, the images were most frequently taken at sites B and C. At each of these two sites, eight images surrounding the station were taken towards the sky at 1.5 m high. The top of the photographs faced north. To avoid the interference due to variation in sunlight, the images were generally taken twice a week on overcast days. In total, 576 images were taken during 36 days throughout the observation period. Table 1 lists the measured items and instruments at five observation sites.

**Table 1:** Measured items and instruments at each observation site

Measured items	Instruments	Features	Observation sites
*T*_*a*_	6382OV Davis Temp Station	• Range −25 to 50 °C • Resolution 0.1 °C • Accuracy ±0.5 °C	A and B and C and D and E
RH	6382OV Davis Hum Station	• Range 0–100 % • Resolution 1 % • Accuracy ±3 %	A and B and C and D and E
*V*_*a*_	7911 Davis Anemometer	• Range 0 to 56 m/s • Resolution >0.05 m/s • Accuracy ±0.5 m/s	A and B
*T*_*g*_	Heat Index WBGT Meter (2010SD)	• Range 0 to 80 °C • Resolution 0.1 °C • Accuracy ±0.6 °C	A and B
PAI	Panasonic Lumix DMC-FZ100 digital camera with a fisheye wide-angle lens (VLB1658B)	–	B and C

### Statistical tests

We first investigated whether the microclimatic data differed among the five observation sites. Since the daily maximum, minimum, and average *T*_*a*_, RH, and *V*_*a*_ were not normally distributed, the statistical significance for differences in these values among the observations sites were analyzed by a non-parametric KruskaleWallis *H* test. However, this test might be obscured by the daily fluctuations of temperature and humidity that were higher than the differences among the sites (especially during spring and summer). To circumvent this problem, we calculated the mean value of all observation sites and used this to determine the relative microclimatic data at each station. The difference between the mean value and the data *x* at each station was given as *D*($x$). This resulting relative value for the daily maximum, minimum, and average is calculated for *T*_*a*_, RH, and *V*_*a*_ as follows:

1 $$ D{(x)}_{dj}={ \max}_i/{ \min}_i/{\mathrm{average}}_i\left({x}_{dji}\right)-\frac{1}{M}{\displaystyle \sum_{j=1}^M}{ \max}_i/{ \min}_i/{\mathrm{average}}_i\left({x}_{dji}\right) $$

where D($x$) denotes the relative daily maximum, minimum, or average value for Ta, RH, or Va and $x$_*dji*_ stands for Ta, RH, or Va data, with the different indices and parameters defined as follows:

*j*: Index of observation site (*j* = 1,…, *M*), *M* = 5
*M*: Total number of observation sites
*d*: Index of observation day (*d* = 1,…, *K*), *K* = 134 days
*K*: Total number of observation days
*i*: Index of data point in 1 day (*i* = 1,…, *N*), *N* = 144
*N*: Total number of data points in 1 day

Subsequently, a KruskaleWallis *H* test was performed on *D*($x$) where $x$ represents either *T*_*a*_, RH, or *V*_*a*_ and with the observation sites as the independent variable. A *p* value of <0.05 indicates that the differences of the dependent variables among the observation sites are significant.

### Thermal comfort level

#### Mean radiant temperature

Apart from understanding the effects of the vegetation on the microclimate, this study also investigates the influence of vegetation on thermal comfort. The mean radiant temperature (Tmrt), as the most influential factor that determines outdoor thermal comfort, was derived for the open space (site A) and the grove (site B). There exist two common methods to calculate Tmrt. The first method is the six-direction radiation method, in which Tmrt is calculated according to the short-wave and long-wave radiation in six directions (VDI 1998). The second method, which is the called the globe thermometer method, calculates Tmrt from Tg, Ta, and Va using the standardized Tmrt equation from (Eq. 2) ISO 7726 (1998). In this study, we used the second method.

2 $$ \mathrm{Tmrt}={\left[{\left({T}_g+273.15\right)}^4+\frac{3.42\times {10}^9{V_a}^{0.119}}{\varepsilon {D}^{0.4}}\times \left({T}_g-{T}_a\right)\right]}^{0.25}-273.15 $$

*T*_*g*_: Globe temperature (°C)
*V*_*a*_: Air velocity (ms^−1^)
*T*_*a*_: Air temperature (°C)
*D*: Globe diameter (m)
*ε*: Globe emissivity

where the globe diameter of the heat index WBGT Meter (*D*) is 75 mm and globe emissivity (*ε*) is normally assumed as 0.95 (Taleghani et al. 2013). *T*_*a*_, *T*_*g*_, and *V*_*a*_ were based on the measurements at sites A and B with a 10-min sample interval.

#### Thermal comfort indices

In order to assess outdoor comfort, or its perception and sensation, various indices combining microclimatic data and biometeorological factors have been developed. A predicted mean vote (PMV) approach is often used to quantify thermal comfort. PMV ranges from −3.5 (very cold) to 3.5 (very hot) (Fanger 1972). Physiological equivalent temperature (PhET) was regularly used in recent studies (Peng and Jim 2013; Abdel-Ghany et al. 2013; Klemm et al. 2014). PhET is equivalent to the *T*_*a*_, with a widely known unit (°C). Hence, PhET is more intuitive and comprehensive compared to PMV. Both indices can be simply calculated by RayMan software (RayMan stands for “radiation on the human body”) (Matzarakis et al. 2007). It requires the following inputs: *T*_*a*_, RH, *V*_*a*_, Tmrt, and the parameters that describe the heat exchange processes of the human body (personal data, clothing, and activity).

### Estimation of plant area index

#### Cap fraction analysis

Leaf area index (LAI) is the ratio of single-sided leaf area (m^2^) to ground surface area (m^2^) (Watson 1947). This is a key parameter to estimate the canopy architecture and ecosystem processes. In the past decades, numerous methods have been developed to quantify LAI directly and indirectly. The direct measurement consists of harvesting the vegetation, applying the allometric equations and collecting leaf litter fall (Wilson 1963; Nizinski and Saugier 1988; Mäkelä et al. 1995; Maguire and Bennett 1996; Thomas and Winner 2000). Although these methods are accurate, they are both destructive and time and labor consuming (Gower et al. 1999). Therefore, many new studies have estimated LAI indirectly. One of these methods is gap fraction analysis that infers the LAI by measuring the light transmittance of the canopy (Smith et al. 1991; Welles and Cohen 1996; Cutini et al. 1998; Stadt and Lieffers 2000; Finzel et al. 2012). This method is based on the Beer-Lambert law for light attenuation through a canopy and requires measurements of above and below canopy radiation. Ideally, the foliage is randomly distributed in the canopy. However, this is generally not the case in reality (Bréda 2003). Hence, the gap fraction depends on both LAI and leaf inclination distribution (Bréda 2003). In this study, we applied the hemispherical photography method to estimate the canopy structure and light transmittance. Firstly, upward-oriented fisheye photographs were taken at eight locations surrounding each observation station. CAN-EYE V6.3 image analysis software (used to extract the canopy structure characteristics from true color images) was used to analyze the resulting images. We then generated several canopy structure characteristics including LAI, average leaf inclination angle, fraction of absorbed photosynthetically active radiation, vegetation cover fraction, and bidirectional gap fraction. The resulting LAI could be overestimated in our study, because stem and branch areas (WAI) are included in this method. CAN-EYE V6.3 supports the masking of parts of the image, but neglects the leaf areas behind these elements and likely underestimates LAI. For this reason, CAN-EYE V6.3 outputs correspond to the plant area index (PAI) that include the ratio of the sum of stems, branches or trunk, and leaf area (m^2^) to ground surface area (m^2^) (Weiss and Baret 2010; Zhao et al. 2012).

#### Regression model

**Fig. 2:**
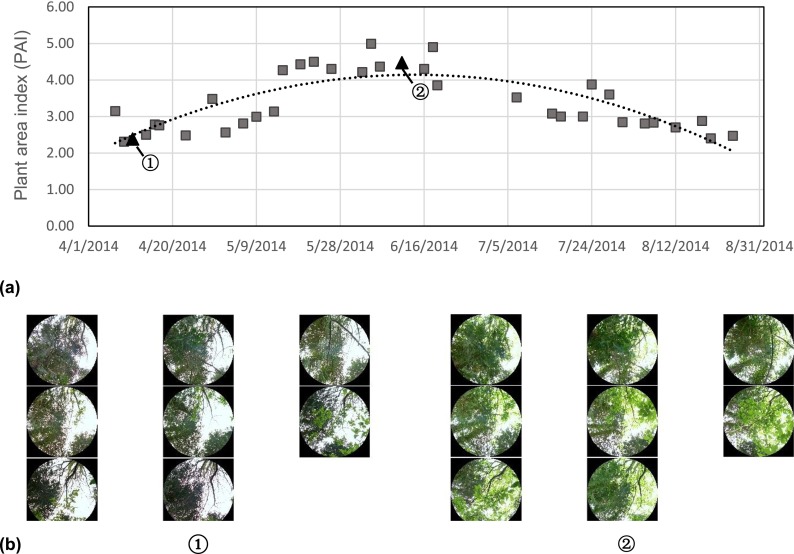
**a** The regression curves derived from the observed PAI in the grove (site B). **b** The examples of sky-view images in leafless season (on 11 March 2014) (*1*) and in full leaf season (on 11 June 2014) (*2*)

**Fig. 3:**
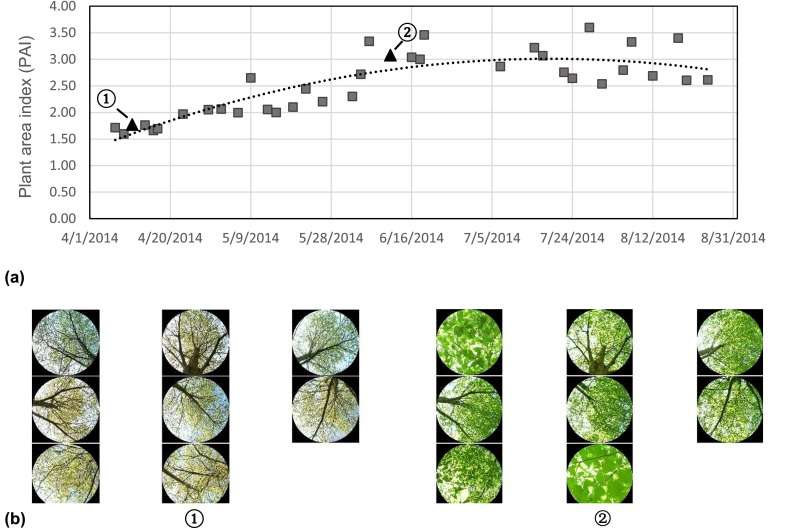
**a** The regression curves derived from the observed PAI beneath a single deciduous tree (site C). **b** The examples of sky view images in leafless season (on 11 March 2014) (*1*) and in full leaf season (on 11 June 2014) (*2*)

PAI values on each day during the observation period were estimated from the measured PAI at sites B and C using a regression model. We found that quadratic equations provided the best fit to the collected data, as theoretically expected for the variation of PAI in this period.

3 $$ \mathrm{Site}\kern0.5em \mathrm{B}:\mathrm{P}\mathrm{A}\mathrm{I}=-0.0004{x}^2+0.154x-8596\left(r=0840\right) $$

4 $$ \mathrm{Site}\ C:\mathrm{P}\mathrm{A}\mathrm{I}=-0.0001{x}^2+0.055x-2592\left(r=0.801\right) $$

where *x* stands for Julian day numbers. Figures 2 and 3 show the regression curves derived from the measured PAI, as well as two examples of sky-view images in leafless and full leaf seasons for sites B and C, respectively. The *R*-square for the two relationships were 0.706 and 0.642, respectively, while the adjusted *R*-square were 0.685 and 0.617. This indicates that the two linear models fit the sets of observations. Using the predicted PAI from Eqs. 3 and 4, we calculated the monthly average PAI. Subsequently, variation of monthly average PAI was compared with the changes of differences in *T*_*a*_ (∆*T*_*a*_) and Tmrt (∆Tmrt) between shaded areas and unshaded area. Afterwards, we examined how the time-varying PAI affected the differences of *T*_*a*_ (∆*T*_*a*_) between shaded and unshaded area. The relationship between predicted PAI and the differences of Tmrt (∆Tmrt) between sites A and B was also estimated.

### Clustering weather conditions

#### Definition of clusters

Our previous study employed a cluster method which integrates the clearness index (Kt), the fluctuation of solar radiation (FR), and the maximum air temperature (MaxTa). This method characterized the weather conditions during the summer and winter observation periods individually (Wang et al. 2015). Kt and FR helped to classify the weather conditions during the observation period via calculating the average solar insolation and determining the fluctuation rate of diurnal solar radiation. Meanwhile, MaxTa highlighted the most uncomfortable days in summer and winter (hottest and coldest days). The current study aims to quantify the changes in the different green infrastructures’ effects on the local urban microclimate from April to August. As using MaxTa for clustering would only highlight the uncomfortable days in the hottest months (July and August), it was not required in this study. Hence, only Kt and FR were used to characterize the weather conditions. Kt is the ratio of the global solar radiation measured at the surface and the clear sky solar radiation, which was proposed by Kuye and Jagtap (1992). Kt, calculated according to the hourly average solar insolation, does not reflect the variation of solar intensity. To capture this, we calculated the fluctuation rate of diurnal solar radiation (FR) to compute the variation of sunlight intensity for a given period (for a 1-min time interval). Since Kt ranges from 0 to 1, FR was transformed to the same scale by applying unity-based normalization. According to the classification of Kt by Liu and Jordan (1960) and our earlier study (Wang et al. 2015), we defined the days having Kt ≥ 0.65 and FR ≤ 0.5 as clear days and the days with Kt < 0.35 and FR ≤ 0.5 as cloudy days, and the remaining days were the near-cloudy or near-clear days.

#### Cluster results

**Fig. 4:**
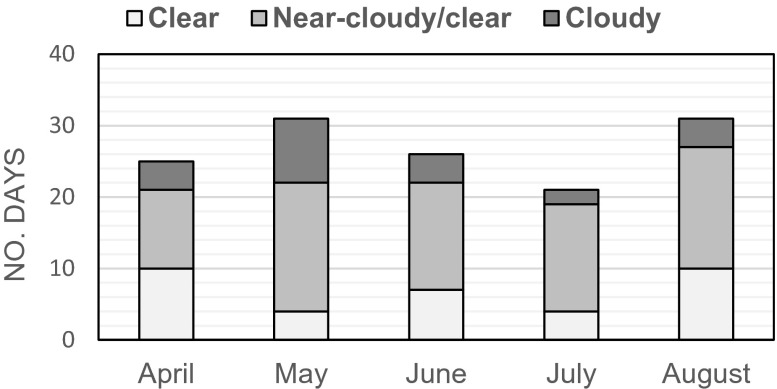
Clustering results for the weather conditions during the observation days from April to August 2014

The cluster results for the weather conditions from April to August 2014 over 134 sampling days are illustrated in Fig. 4. Notably, the days with incomplete data were eliminated from the total sampling days. In total, there were 23 cloudy days and 35 clear days in the observation period. The rest 76 days were near-cloudy or near-clear days. The clustering approach classified the microclimatic effects of green infrastructure under different weather conditions. Additionally, KruskaleWallis significance test for the differences of microclimatic data for different weather conditions was performed for each month.

## Results

### Microclimatic differences

As far as (*x*) (differences in *T*_*a*_, RH, and *V*_*a*_ between each observation site and the mean value) was concerned, the KruskaleWallis *H* test revealed significant differences with *p* < 0.0005 during the daytime. Comparisons among multiple observation sites showed that *D* (daily maximum, average, minimum *T*_*a*_) of site A differed significantly from the other sites (*p* < 0.0005 for all). However, the differences of *D* (maximum, average, minimum RH) between sites A and D were not significant with *p* > 0.05 for all. Similar results were also found during the nights.

**Fig. 5:**
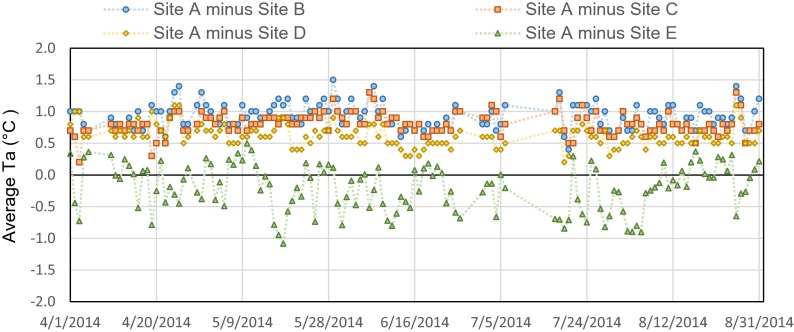
Differences of daily average *T*_*a*_ during the daytime. Site A—open space. Site B—grove. Site C—single deciduous tree. Site D—street trees. Site E—building façade

Figure 5 depicts the differences of daily average *T*_*a*_ between the unshaded area (site A) and the four shaded areas by trees (sites B, C, and D) or buildings (site E). As expected, site A always had higher daily average *T*_*a*_ compared to sites B, C, and D. During the whole observation period, daily average *T*_*a*_ in the three shaded areas were respectively 0.9 °C (standard deviation (SD) = 0.2 °C), 0.8 °C (SD = 0.2 °C), and 0.6 °C (SD = 0.1 °C) lower, compared to unshaded area. These differences in daily maximum *T*_*a*_ between the shaded and unshaded areas increased to 1.9 °C (SD = 0.5 °C), 1.6 °C (SD = 0.4 °C), and 1.4 °C (SD = 0.4 °C). All these differences were larger than the accuracy of the instruments (>0.5 °C). On hot and dry days, the maximum temperature differences could reach 3.3, 2.7, and 2.3 °C for the tree-shaded sites, respectively. These results showed that the grove site had the best cooling capability compared to the other sites, although the difference was rather small. Figure S1 gives the observed range of *T*_*a*_ differences and the average difference over a single day during the observation period. Moreover, we found that, compared to the unshaded area, the building façade remarkably increased the daily maximum *T*_*a*_ by 1.6 °C (SD = 1.0 °C). This difference peaked (3.5 °C) on 19th May. However, the shade of the building could also decrease the daily average *T*_*a*_ by as much as 0.5 °C.

In terms of RH, the grove (site B) had the highest daily average RH (76 %); site E, which was adjacent to the building façade, presented the lowest daily average RH (72 %). However, the SDs of average RH of all five sites (9–10 %) were much bigger than the differences among them. This indicates that RH was not varying much in the research area and that trees have a rather limited effect on the RH distributions. As described in the above paragraph, no statistically significant difference in RH between sites A and D was found (average RH = 75 % for both sites). Tables that summarize the differences of daytime *T*_*a*_ and RH between unshaded and the shaded areas are shown in Tables S1 to S4.

Theoretically, *T*_*a*_ in open spaces decreases more markedly and quickly than in tree-shaded areas after sunset (Akbari 2002). Hence, this effect enlarges the *T*_*a*_ range and its rate of change. Although the differences of *T*_*a*_ and RH were significant, the expected higher *T*_*a*_ range and its rate of change in the open space were not observed. In addition, we found that the *V*_*a*_ significantly influenced the nightly *T*_*a*_ trends among the observation sites (*p* < 0.0005).

**Fig. 6:**
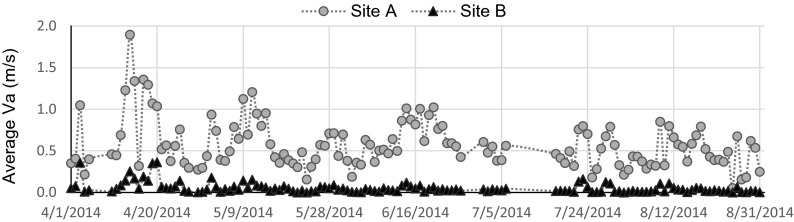
The average wind velocity at sites A and B

The wind measurements were taken simultaneously in the open space and in the grove. Figure 6 shows that the average *V*_*a*_ in the open space significantly differed from that in the grove (*p* < 0.0005). The daily maximum and average *V*_*a*_ in the grove were 0.8 m/s (SD = 0.4 m/s) and 0.1 m/s, whereas these values in the open space were approximately 2.8 m/s (SD = 1.1 m/s) and 0.6 m/s (SD = 0.3 m/s). That is to say, trees as shelter lowered the wind speed dramatically.

### Thermal comfort level in daytime

The values of mean radiative temperature (Tmrt; Eq. 2) at sites A and B were calculated. The result showed that the measurements in the open space exhibited higher Tmrt values when compared with the measurements in the grove, being 7.4 °C (SD = 2.0 °C) and 11.5 °C (SD = 2.5 °C) higher for the average and maximum Tmrt, respectively.

**Fig. 7:**
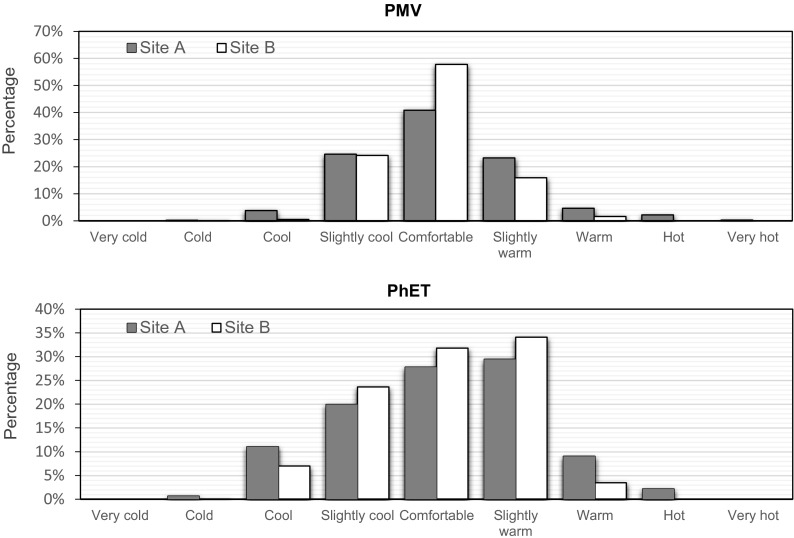
Percentage of diurnal PMV (*top*) and PhET (*bottom*) value for the different thermal perception in the open space (site A) and in the grove (site B)

After analyzing the Tmrt differences between sites A and B, we analyzed the diurnal ranges in these two areas through calculating the thermal comfort indices (predicted mean vote (PMV) and physiological equivalent temperature (PhET)). Both PMV and PhET values are expressed as a percentage of thermal perception (Fig. 7). The results showed that trees drastically improve comfort, resulting in higher percentage of “comfortable” perception and lower percentage of “hot” and “cool” perception. Taking, for example, one of the hottest days (18th July), during the hottest afternoon hours, open space experienced around 4 h of hot period. In the grove, however, “slightly warm” and “warm” thermal perception sustained during the same time period, while hot sensation was absent. Figure S2 lists these diurnal PhET and PMV values from 10:00 to 18:00 on 18th July.

### Plant area index effects

**Fig. 8:**
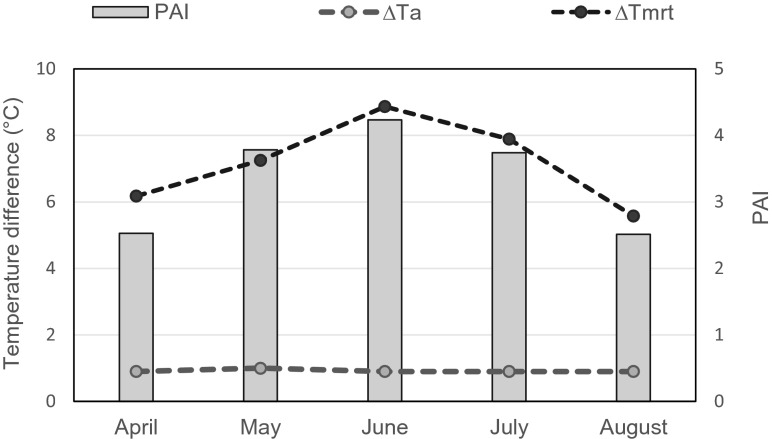
The variation of monthly average PAI, ∆*T*_*a*_, and ∆Tmrt

The average values of PAI, ∆*T*_*a*_, and ∆Tmrt (between the shaded areas and unshaded area) were calculated for each month. Figure 8 depicts the variation of monthly average PAI, ∆*T*_*a*_, and ∆Tmrt. It can be observed that monthly average ∆*T*_*a*_ has not undergone much variation during the growing season. In contrast, ∆Tmrt shows a sensitivity to the changes of PAI. Starting from April, the monthly average ∆Tmrt gradually increased and ended up at 8.9 °C in June, when the average PAI reached a peak level of 4.2. Subsequently, both values declined simultaneously. This behavior revealed that most probably, the changes in monthly average ∆Tmrt follow the same pattern of the variations in average PAI.

To better understand the relationship between PAI and temperature reduction by green infrastructure, a linear regression analysis was applied between the predicted PAI towards the differences in daily average *T*_*a*_ and Tmrt. The simple correlation equations with 95 % confidence limits are as follows:

5 $$ \varDelta {T}_a=0.696+0.059\mathrm{P}\mathrm{A}\mathrm{I}\left(r=0.237\right) $$

6 $$ \varDelta \mathrm{Tmrt}=0.097+2.054\mathrm{P}\mathrm{A}\mathrm{I}\left(r=0.655\right) $$

**Fig. 9:**
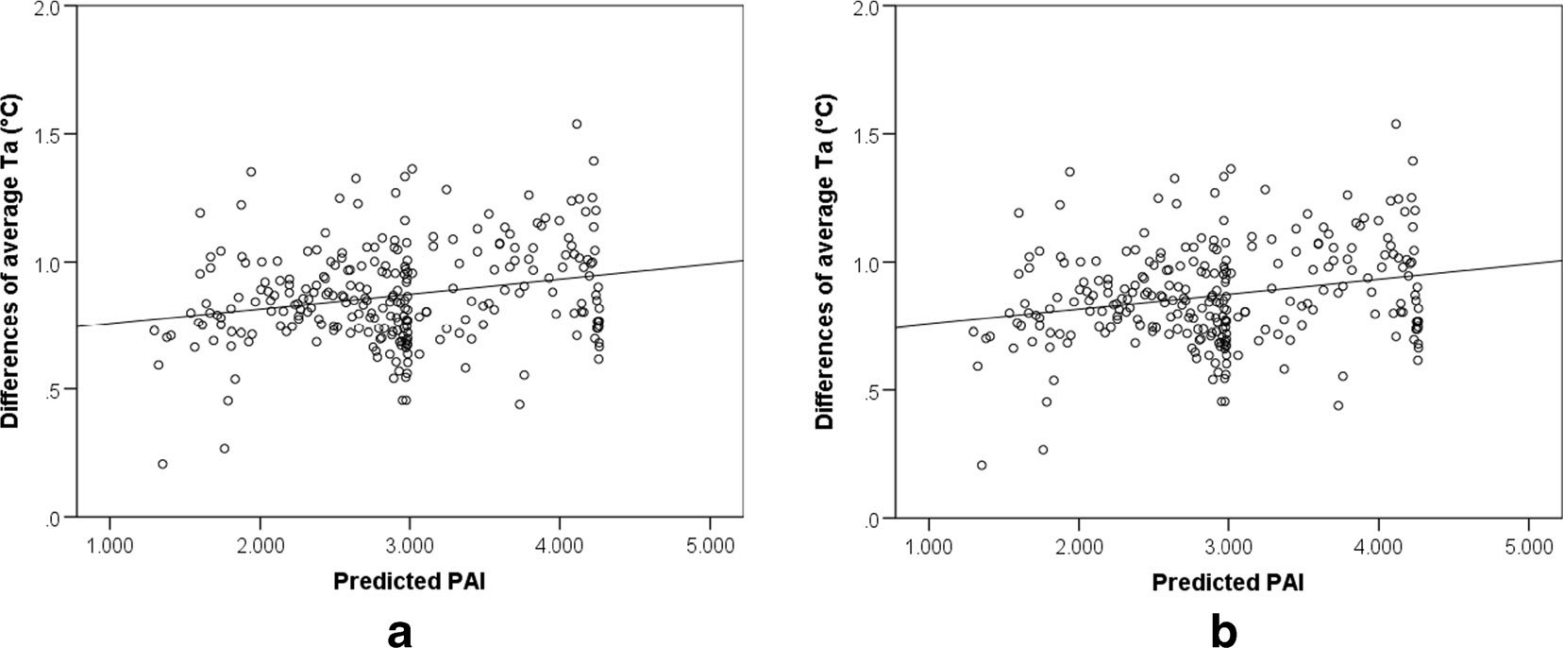
Scatter plots and linear-fit estimation of ∆*T*_*a*_ vs. PAI (**a**) and ∆Tmrt vs. PAI (**b**)

The scatter plots in Fig. 9 show the linear-fit estimation of ∆*T*_*a*_ and ∆Tmrt with the PAI. The values of the *t* statistic on the coefficients of these two linear regressions were 3.980 and 3.975, respectively, while both *p* values were less than 0.05. These results indicate that the resulting coefficients are significant and the variations explained by the models are robust. However, the gradient coefficient of PAI for the temperature regression equation was only 0.059. In other words, a gain in PAI value of one unit would result in a ∆*T*_*a*_ increase of 0.059 °C, which is smaller than the measurement accuracy (±0.5 °C). Therefore, this linear relationship seems unreliable. The variation in PAI was found to have a stronger correlation with the ∆Tmrt changes (c.f. the gradient coefficient of 2.1). This is likely due to the fact that, compared to *T*_*a*_, Tmrt is more strongly affected by trees (Matzarakis et al. 1999). Regarding the thermal comfort level, the monthly average PAI of trees in the grove reached their highest value in June and this led to more comfortable thermal perception by 14 % compared to open space.

### Weather effects

**Fig. 10:**
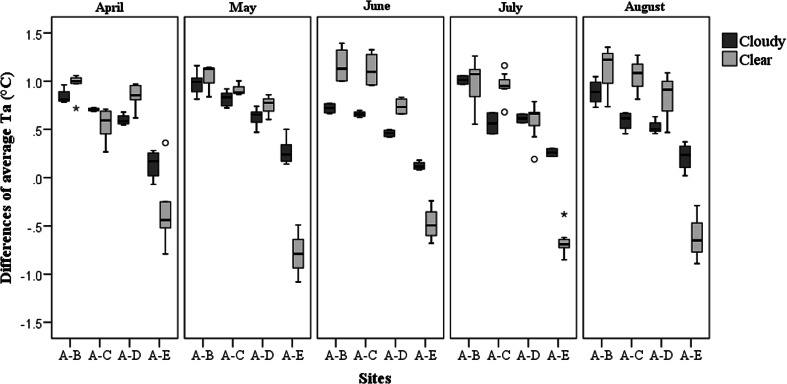
Box plot of ∆*T*_*a*_ for site A minus site B, site A minus site C, site A minus site D, and site A minus site E on cloudy and clear days from April to August 2014

Figure 10 compares ∆*T*_*a*_ (for site A minus site B, site A minus site C, site A minus site D, and site A minus site E) between daytime cloudy days and clear days. The weather condition was a significant factor explaining the differences in daily average *T*_*a*_ between tree-shaded areas (sites B, C, and D) and the unshaded area (site A) (*p* < 0.05 for all). It also explained the difference of daily average *T*_*a*_ between the open site A and site E (adjacent to the building façade) (*p* < 0.0005). When performing a KruskaleWallis *H* test for each month, as expected, the weather conditions had a significant impact on the differences of daily average *T*_*a*_ between tree-shaded areas and the unshaded area, but only during the period that trees had a high PAI (summer months). In contrast, weather conditions always played a crucial role in defining the difference of daily average *T*_*a*_ between sites A and E during the entire observation period (*p* < 0.05 for every month). Notably, on the cloudy days, the daily average *T*_*a*_ adjacent to the building (site E) was lower than the open space (site A), whereas on clear days, the values were opposite. Most likely, the building façade absorbs incident solar radiation and releases heat to the ambient environment.

To account for the weather effects during full leaf season, we compared the difference of *T*_*a*_ between the open space and the grove for different weather types from June to August. On cloudy days, the trees in the grove reduced daily maximum and average *T*_*a*_ by about 1.6 °C (SD = 0.3 °C) and 0.8 °C (SD = 0.1 °C), while on clear days, the temperature reductions were 2.2 °C (SD = 0.4 °C) and 1.1 °C (SD = 0.2 °C). For the cooling effects of the single tree at site C, the daily maximum and average *T*_*a*_ were 1.2 °C (SD = 0.2 °C) and 0.6 °C (SD = 0.1 °C) lower than those in the open space under cloudy weather conditions. However, on clear days, the differences increased to 2.2 °C (SD = 0.3 °C) and 1.0 °C (SD = 0.2 °C) for daily maximum and average *T*_*a*_, respectively.

## Discussion

### Microclimatic differences

Based on our measurements, we determined the ranking for the cooling capability of different green infrastructures: grove (site B) > single deciduous tree (site C) > street trees (site D). This confirms a model-based study by Shahidan et al. (2012) who showed that a large number of trees reduced temperature more, although we found that the ground surface also contributes to a *T*_*a*_ reduction. Our study found that the grove with the most trees yields the best cooling capability but that a single tree has a larger cooling effect than a group of street trees, probably due to the paved surface that offsets (some of) the temperature reduction. Furthermore, we also found that the difference in RH between open space and street trees was not significant. A plausible explanation is that both locations were adjacent to the street. RH’s value and trend were therefore significantly influenced by the paved surfaces. In addition, the expected higher *T*_*a*_ range and rate of change at night in the open space were not observed. *V*_*a*_ was found a significant factor in explaining the *T*_*a*_ trend. Hence, heat convection likely plays the most important role at night in small urban areas.

### Thermal comfort indices

Both estimated PMV and PhET values based on the RayMan model confirmed that trees in the grove improved human comfort levels. However, the results of PMV and PhET showed a slightly different frequency pattern (c.f. Fig. 7) because these two indices are derived from different models and approaches. PMV is based on Fanger’s model (Fanger 1972) and calculates the energy loss from the body by determining the skin temperature and evaporative sweat rate. The heat transfer through and from a body is assumed to be at steady state with the environment. PhET estimates the thermal sensation and the corresponding heat stress based on the Munich energy balance model for individuals (MEMI) (Höppe 1993), which calculates the thermal conditions of the body by integrating surrounding meteorological parameters and the physical activity and the clothing of the individual. PhET is not constrained by a steady state heat balance approach.

### Plant area index

To exclude the radiation that is reflected or transmitted by leaves from the observed radiation, upward-oriented fisheye photographs must be taken under diffuse light conditions (cloudy days or twilight periods on sunny days; Zhao et al. 2012). Although we selected the cloudy days to obtain these images, Dutch daily weather changes quickly, leading to abrupt changes in solar intensity and this probably causes the deviation between the true value and the measured value. Shahidan et al. (2012) compared the cooling performance of trees with high (LAI = 9.7) and low (LAI = 0.9) canopy densities in an ENVI-met model. Their result showed that air temperature increased by 0.034 °C when LAI decreases 1 integer unit. This is slightly less than our finding (0.059 °C increase in ∆*T*_*a*_). However, the slope coefficient of the regression line was smaller than the accuracy of measurements. This linear regression relationship is probably unreliable. To study the effects of changes in PAI (or LAI) on air temperature, future studies will require a temperature sensor with better resolution and accuracy. Additionally, in this linear model, PAI was the only independent variable. There might be other factors that could mimic or perhaps obscure the effects of PAI on ∆*T*_*a*_. A comprehensive relationship between ∆*T*_*a*_ and possible factors should be explored in future studies.

## Conclusion

This empirical study reports on the effect of different types of urban green infrastructure (UGI) on the microclimate and thermal conditions during the growing season in a local urban area based on actual weather measurements and estimated human thermal perception. From April to August, microclimate data were acquired at five different locations: an open space, a grove, a single deciduous tree, street trees, and a building façade.

The results showed that the grove (with most trees) had the best cooling capability among the studied types of UGI although the difference in the daily average air temperature (*T*_*a*_) reduction was small (max 0.3 °C). In addition, lower wind velocity (*V*_*a*_) values were also found in the grove. The effects of these meteorological changes on human thermal comfort were confirmed through estimating the perception of thermal comfort, which allowed to establish a quantitative relationship between different types of UGI, their microclimate effects, and influence on human thermal perception. Another finding was that street trees performed worse in terms of microclimate regulation than a single tree, probably due to the presence of paved surfaces which offset temperature reductions. Furthermore, the *T*_*a*_ differences between the open space and the area adjacent to the building façade fluctuated (the *T*_*a*_ differences could be both positive and negative) due to the weather conditions and the direction of the sun. The weather conditions were also a significant factor in explaining the *T*_*a*_ differences between open space and the other tree-shaded areas. In general, the cooling effect of the trees on clear days was shown to be almost two times higher than on cloudy days. Changes of monthly average ∆Tmrt followed the same pattern of the variations in monthly average PAI, with a separated peak level of 8.9 °C and 4.2 in June. Through linear regression analysis, the relationship between ∆*T*_*a*_ and plant area index (PAI) was determined. The slope coefficient of PAI for the regression equation was too small to explain the changes in ∆*T*_*a*_, but the differences in ∆Tmrt between the open space and the grove were significantly related to PAI (gradient coefficient = 2.054).

Increasing the PAI through appropriate UGI measures may thus considerably reduce Tmrt and enhance thermal comfort locally during spring and summer. In order to verify and further quantify this finding, the effects of changes in PAI need to be explored further in a more comprehensive regression model supported by empirical data on climate and human perception. It should thereby be realized that weather conditions have a notable effect on the climate-regulation performance of green infrastructure.

## Electronic supplementary material

Fig. S1 (PDF 161 kb)

Fig. S2 (PDF 272 kb)

Table S1 (PDF 105 kb)

Table S2 (PDF 104 kb)

Table S3 (PDF 104 kb)

Table S4 (PDF 105 kb)
