# Supplementary material for: Effects of urban green infrastructure (UGI) on local outdoor microclimate during the growing season
Source: Environ Monit Assess. 2015 Nov 7;187:732. doi: 10.1007/s10661-015-4943-2 (PMC4636989; doi:10.1007/s10661-015-4943-2)
Supplement: Supplementary file 1 — (PDF 161 kb) [file 10661_2015_4943_MOESM1_ESM.pdf]

Supplementary material

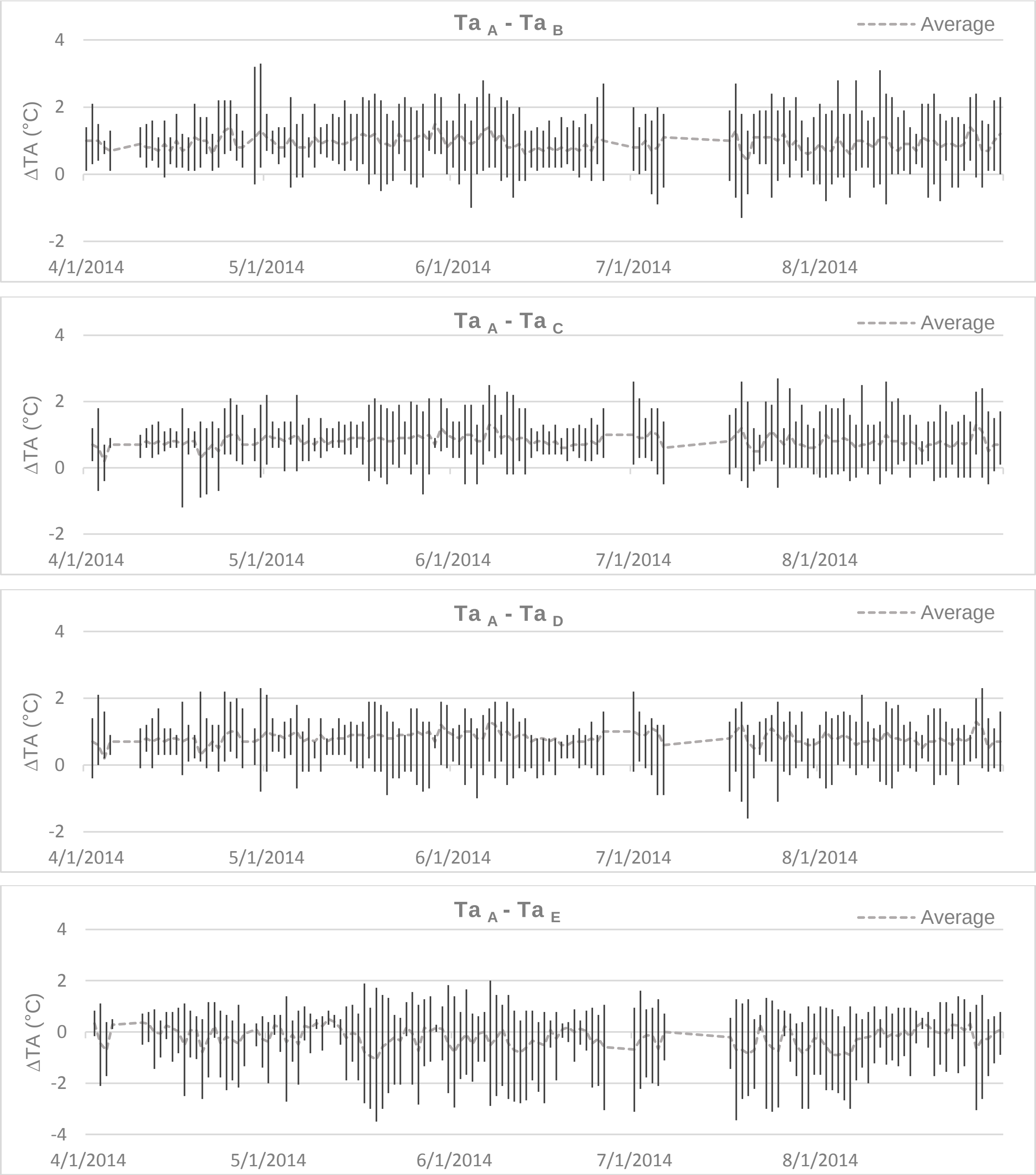

Fig. S1 Ta differences between Sites A and B; Sites A and C; Sites A and D; and Sites A and E

Note: The vertical line on the chart shows the range of Ta differences (the highest and lowest values) over one day
