# Supplementary figures and images for: Effects of urban green infrastructure (UGI) on local outdoor microclimate during the growing season
Source: Environ Monit Assess. 2015 Nov 7;187:732. doi: 10.1007/s10661-015-4943-2 (PMC4636989; doi:10.1007/s10661-015-4943-2)

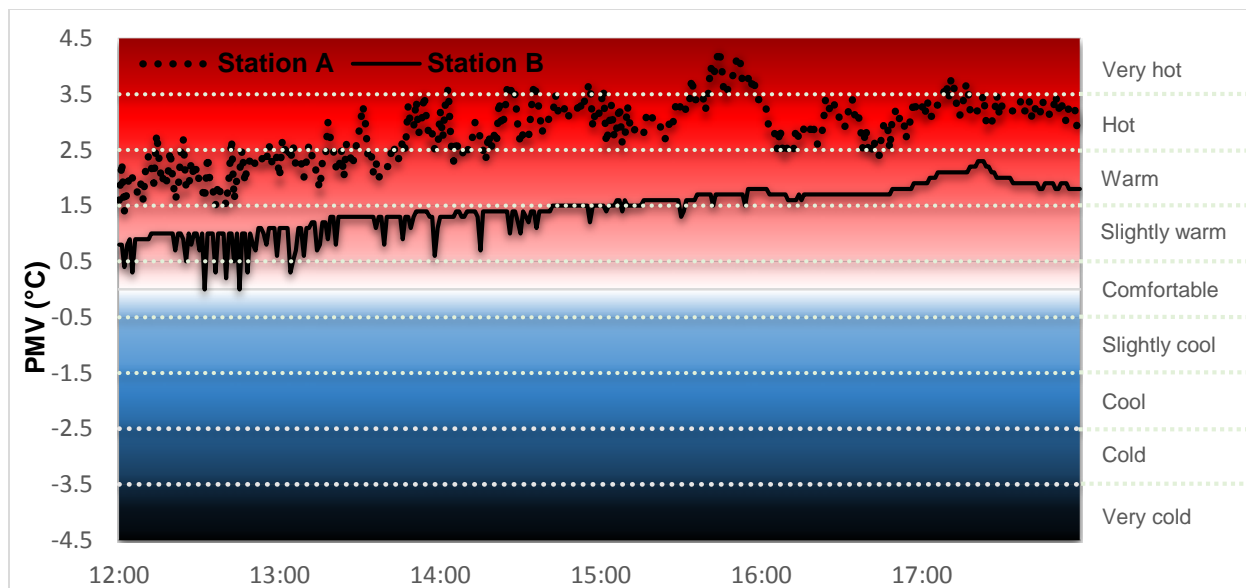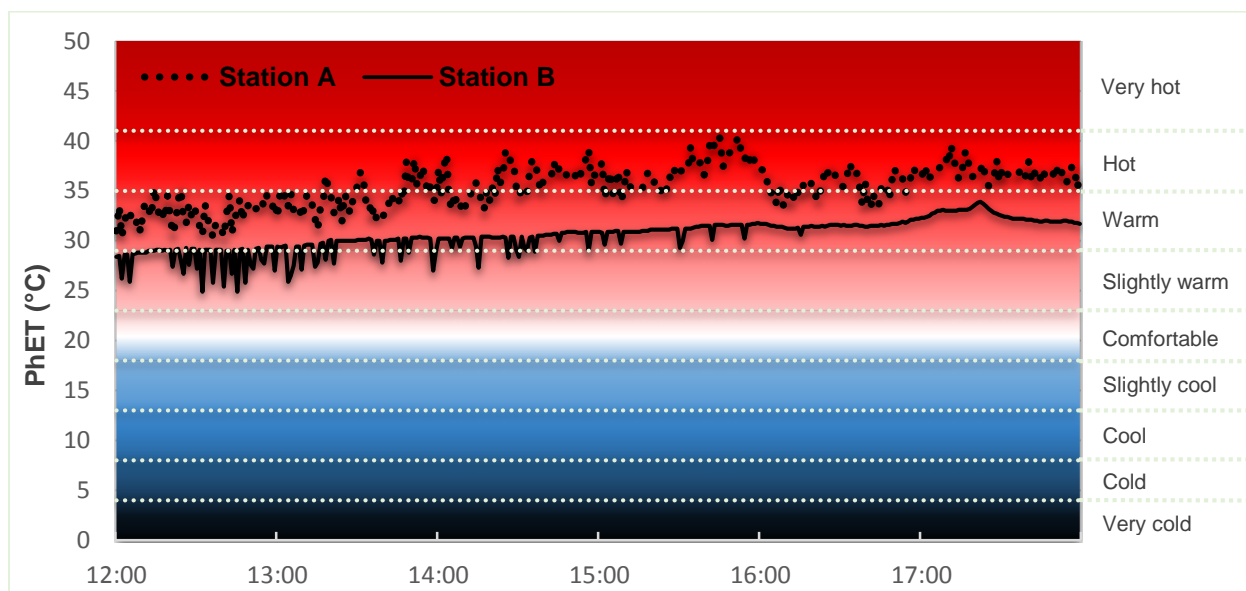

**Fig. S2** Diurnal PhET and PMV values from 10:00 to 18:00 on 18<sup>th</sup> July

Supplement: Supplementary file 2 — (PDF 272 kb) [file 10661_2015_4943_MOESM2_ESM.pdf]
