# Supplementary material for: Effects of urban green infrastructure (UGI) on local outdoor microclimate during the growing season
Source: Environ Monit Assess. 2015 Nov 7;187:732. doi: 10.1007/s10661-015-4943-2 (PMC4636989; doi:10.1007/s10661-015-4943-2)
Supplement: Supplementary file 3 — (PDF 105 kb) [file 10661_2015_4943_MOESM3_ESM.pdf]

Table S1 Summarize table on differences of daytime Ta and RH between Site A (open space) and Site B (grove)

**Sites**	**No. of days** (N)	**Features**	**Differences of Ta (°C)** A - B			**Differences of RH (%)** A - B		
			Value per day	*Mean*	*Std. Deviation*	*Value per day*	*Mean*	*Std. Deviation*
April	25	Maximum	1.0–3.3	1.7	0.6	0–6	2	1
	25	Minimum	-0.3–0.7	0.3	0.2	-15–-1	-5	3
	25	Average	0.6–1.4	0.9	0.2	-4–1	-1	1
May	31	Maximum	1.1–2.4	1.9	0.4	-1–9	3	3
	31	Minimum	-0.5–0.7	0.2	0.4	-10–-3	-6	2
	31	Average	0.8–1.5	1.0	0.2	-3–1	-1	1
June	26	Maximum	1.1–2.8	1.8	0.5	1–8	3	2
	26	Minimum	-1.0–0.3	-0.0	0.3	-9–-2	-5	2
	26	Average	0.6–1.4	0.9	0.2	-2–0	-1	1
July	21	Maximum	1.1–2.7	1.9	0.4	0–11	4	3
	21	Minimum	-1.3–0.6	-0.2	0.5	-9–-2	-5	2
	21	Average	0.4–1.3	0.9	0.2	-3–2	-1	1
August	31	Maximum	1.2–3.1	2.0	0.5	-1–8	3	2
	31	Minimum	-0.9–0.4	-0.2	0.4	-14–-1	-6	3
	31	Average	0.6–1.4	0.9	0.2	-4–2	-1	1
TOTAL	134	Maximum	1.0–3.3	1.9	0.5	-1–11	3	2
	134	Minimum	-1.3–0.7	-0.0	0.4	-15–-1	-5	3
	134	Average	0.4–1.5	0.9	0.2	-4–2	-1	1
