# Supplementary material for: Effects of urban green infrastructure (UGI) on local outdoor microclimate during the growing season
Source: Environ Monit Assess. 2015 Nov 7;187:732. doi: 10.1007/s10661-015-4943-2 (PMC4636989; doi:10.1007/s10661-015-4943-2)
Supplement: Supplementary file 4 — (PDF 104 kb) [file 10661_2015_4943_MOESM4_ESM.pdf]

Table S2 Summarize table on differences of daytime Ta and RH between Site A (open space) and Site B (single deciduous tree)

**Sites**	**No. of days** (N)	**Features**	**Differences of Ta (°C)** A - C			**Differences of RH (%)** A - C		
			Value per day	*Mean*	*Std. Deviation*	*Value per day*	*Mean*	*Std. Deviation*
	25	Maximum	0.7–2.2	1.4	0.4	0–8	3	1
April	25	Minimum	-1.2–0.6	0.1	0.6	-11–1	-4	2
	25	Average	0.2–1.0	0.7	0.2	-3–1	-0	1.1
	31	Maximum	0.9–2.2	1.6	0.4	-1–8	2	2
May	31	Minimum	-0.8–0.6	0.2	0.4	-11–-2	-5	2
	31	Average	0.7–1.2	0.9	0.1	-4–0	-1	1
	26	Maximum	0.9–2.6	1.6	0.5	0–5	3	1
June	26	Minimum	-0.5–0.5	0.2	0.3	-9–-2	-5	2
	26	Average	0.6–1.3	0.8	0.2	-3–1	-1	1
	21	Maximum	1.0–2.7	1.7	0.5	1–9	4	2
July	21	Minimum	-0.6–0.3	-0.1	0.3	-8–-1	-5	2
	21	Average	0.5–1.2	0.8	0.2	-2–2	-0	1
	31	Maximum	0.8–2.6	1.7	0.4	1–6	4	1
August	31	Minimum	-0.5–0.4	-0.1	0.2	-10–-1	-5	2
	31	Average	0.5–1.3	0.8	0.2	-3–2	0	1
	134	Maximum	0.7–2.7	1.6	0.4	-1–9	3	2
TOTAL	134	Minimum	-1.2–0.7	0.1	0.4	-11–-1	-5	2
	134	Average	0.2–1.3	0.8	0.2	-4–2	-1	1
