# Supplementary material for: Effects of urban green infrastructure (UGI) on local outdoor microclimate during the growing season
Source: Environ Monit Assess. 2015 Nov 7;187:732. doi: 10.1007/s10661-015-4943-2 (PMC4636989; doi:10.1007/s10661-015-4943-2)
Supplement: Supplementary file 5 — (PDF 104 kb) [file 10661_2015_4943_MOESM5_ESM.pdf]

Table S3 Summarize table on differences of daytime Ta and RH between Site A (open space) and Site D (street trees)

Sites	No. of days (N)	Features	Differences of Ta (°C) A - D			Differences of RH (%) A - D		
			Value per day	*Mean*	*Std. Deviation*	*Value per day*	*Mean*	*Std. Deviation*
April	25	Maximum	0.9–2.3	1.5	0.5	0–6	3	1
	25	Minimum	-0.8–0.4	0.0	0.3	-10–0	-3	2
	25	Average	0.5–1.1	0.7	0.2	-2–1	-0	1
May	31	Maximum	0.7–1.9	1.4	0.3	-1–8	3	2
	31	Minimum	-0.9–0.5	-0.1	0.4	-7–-2	-3	0
	31	Average	0.4–0.9	0.6	0.1	-2–1	0	1
June	26	Maximum	0.6–2.2	1.3	0.4	1–10	4	2
	26	Minimum	-1.0–0.2	-0.2	0.3	-7–-1	-3	1
	26	Average	0.3–0.8	0.5	0.1	-1–1	0	0
July	21	Maximum	0.8–1.9	1.4	0.3	0–12	4	3
	21	Minimum	-1.6–0.3	-0.4	0.5	-7–-1	-4	2
	21	Average	0.2–0.8	0.5	0.2	-3–2	0	1
August	31	Maximum	0.9–2.3	1.5	0.3	1–8	3	2
	31	Minimum	-0.7–0.2	-0.2	0.3	-8–-2	-4	2
	31	Average	0.4–1.1	0.6	0.1	-2–2	0	1
TOTAL	134	Maximum	0.6–2.3	1.4	0.4	-1–12	4	2
	134	Minimum	-1.6–0.5	-0.1	0.4	-10–0	-3	2
	134	Average	0.2–1.1	0.6	0.1	-3–2	0	1
