# Supplementary material for: Effects of urban green infrastructure (UGI) on local outdoor microclimate during the growing season
Source: Environ Monit Assess. 2015 Nov 7;187:732. doi: 10.1007/s10661-015-4943-2 (PMC4636989; doi:10.1007/s10661-015-4943-2)
Supplement: Supplementary file 6 — (PDF 105 kb) [file 10661_2015_4943_MOESM6_ESM.pdf]

Table S4 Summarize table on differences of daytime Ta and RH between Site A (open space) and Site E (building façade)

Sites	No. of days (N)	Features	Differences of Ta (°C) A - E			Differences of RH (%) A - E		
			Value per day	*Mean*	*Std. Deviation*	*Value per day*	*Mean*	*Std. Deviation*
April	25	Maximum	0.3–1.2	0.7	0.3	3–13	8	3
	25	Minimum	-2.6–0.1	-1.3	0.8	-6–1	-1	1
	25	Average	-0.8–0.4	-0.1	0.3	1–5	3	1
May	31	Maximum	0.3–1.9	1.0	0.4	2–13	8	4
	31	Minimum	-3.5–0.3	-1.5	1.1	-8–2	-3	3
	31	Average	-1.1–0.5	-0.2	0.4	0–6	2	2
June	26	Maximum	0.3–2.0	0.9	0.4	3–13	8	3
	26	Minimum	-3.1–-0.2	-1.9	0.9	-9–0	-2	2
	26	Average	-0.8–0.2	-0.3	0.3	1–4	3	1
July	21	Maximum	0.3–1.6	0.9	0.3	2–12	9	3
	21	Minimum	-3.4–0.1	-2.0	0.9	-5–1	-2	2
	21	Average	-0.9–0.3	-0.4	0.4	0–5	3	1
August	31	Maximum	0.2–1.4	0.9	0.3	2–13	8	3
	31	Minimum	-3.1–0.1	-1.4	0.8	-4–1	-2	1
	31	Average	-0.9–0.4	-0.1	0.4	1–6	3	1
TOTAL	134	Maximum	0.2–2.0	0.9	0.4	2–13	8	3
	134	Minimum	-3.5–0.3	-1.6	1.0	-9–2	-2	2
	134	Average	-1.1–0.5	-0.2	0.4	0–6	3	1
